# Pretreatment BAN Score Based on Body-mass-index, Albumin and Neutrophil-lymphocyte Ratio Could Predict Long-term Survival for Patients with Operable Esophageal Squamous Cell Carcinoma

**DOI:** 10.7150/jca.73347

**Published:** 2022-06-13

**Authors:** Fei Zhang, Li-Jia Bu, Rong Wang, Jie Da, Jin-Xia Ding, Wan-Ren Peng

**Affiliations:** Department of Oncology, the First Affiliated Hospital of Anhui Medical University, Hefei, Anhui, 230022, P. R. China.

**Keywords:** Body-mass-index, Albumin, Neutrophil-lymphocyte-ratio, Esophagectomy, Prognostic marker, Esophageal squamous cell carcinoma

## Abstract

**Background:** The present study was designed to examine the prognostic value of a systemic inflammation marker-BAN score, which was established based on body-mass-index (BMI), albumin (ALB) and neutrophil-lymphocyte ratio (NLR) in resectable esophageal squamous cell carcinoma (ESCC) patients.

**Methods:** A total of 420 newly diagnosed ESCC patients in our hospital between January 2008 and December 2013 were included. Their baseline characteristics were retrospectively reviewed and collected. BAN score was calculated as (BMI × ALB/ NLR). The optimal cutoff value for BAN score was defined as 28.0 in terms of survival. Patients were then allocated to high BAN (≥ 28.0) and low BAN (< 28.0) score groups.

**Results:** Pretreatment BAN score was significantly associated with tumor length, white blood cell count, BMI, ALB and NLR levels. However, no significant difference was observed in patients' age, gender, tumor location, degree of differentiation, depth of invasion, lymph node involvement, tumor-node-metastasis (TNM) stage or other variables between groups. Moreover, those with high pretreatment BAN scores (≥ 28.0) tended to have favorable disease free survival (DFS) [hazard ratio (HR), 0.650; 95% confidence interval (CI), 0.481-0.877; *P* = 0.005] and overall survival (OS) (HR, 0.608; 95% CI, 0.445-0.829; *P* = 0.002) by univariate analysis. Furthermore, multivariate Cox regression analysis suggested that high BAN score (≥ 28.0) could serve as an independent predictor for both DFS (HR, 0.726; 95% CI, 0.532-0.993; *P* = 0.045) and OS (HR, 0.670; 95% CI, 0.485-0.927; *P* = 0.016).

**Conclusions:** Pretreatment BAN score could independently predict long-term survival for resectable ESCC patients.

## Introduction

Esophageal cancer (EC) is one of the most commonly seen malignancies and the fourth leading cause of cancer-related death in China 1. Unlike Western countries, more than 90% of EC cases are classified as esophageal squamous cell carcinoma (ESCC) in Chinese population 1-3. And approximately two thirds of the patients are diagnosed with advanced or metastatic diseases at first presentation. Although great advances have been achieved in early screening, diagnosis and treatment for the last two decades, ESCC remains one of the most deadliest malignant diseases, with a reported 5-year survival rate of about 30% 1.

Results from previous studies have confirmed that systemic inflammation response plays crucial roles in the process of tumor development, progression and metastasis 4-5. Additionally, a broad range of cancer related symptoms including anorexia, fatigue and cancer cachexia have also been recognized to be attributable to systemic inflammation response 6. Furthermore, the degree of systemic inflammation response has been reported to be significantly associated with impaired survival in cancer patients 4, 7.

Recently, several systemic inflammation-based prognostic variables such as modified Glasgow prognostic score (GPS), hypoalbuminaemia, neutrophil-lymphocyte ratio (NLR) and platelet-lymphocyte ratio (PLR) 8-10 have been established to help risk classification and optimal treatment for ESCC. On the one hand, hypoalbuminemia has been suggested not only as an indicator of poor nutritional condition but also significantly correlated with chronic inflammation in cancer patients 11, and it could also predict worse survival for ESCC cases 12-13. On the other hand, as a novel and promising blood marker of systemic inflammation response, an elevated NLR has been shown to be associated with a higher risk of postoperative recurrence and poor prognosis in various malignancies, including ESCC 14-15. Most recently, Arigami T, et al. found that preoperative NLR was a valuable indicator for predicting disease progression and prognosis in ESCC subjects who received esophagectomy with lymphadenectomy 16. Furthermore, Sun and his colleagues suggested that BMI could be used as a prominent marker for evaluation of nutritional status and a low BMI was an independent indicator for unfavorable OS in Chinese patients with ESCC 17.

Therefore, we proposed a novel and promising inflammation index-BAN score based on a combination of patient's BMI, ALB and NLR to reflect the degree of systemic inflammation response in resectable ESCC. However, its clinical significance has not yet been determined. The purpose of this study was to examine if pretreatment BAN score could predict long-term survival in patients with operable ESCC.

## Patients and methods

### Patients

A cohort of 420 newly and histopathologically diagnosed ESCC patients who underwent esophagectomy with lymphadenectomy at the Department of Thoracic Surgery, the First Affiliated Hospital of Anhui Medical University between January 2008 and December 2015 were selected in the present study. Patients who were diagnosed with malignancies other than ESCC, who received neoadjuvant chemotherapy and/or radiotherapy and those who were diagnosed with autoimmune disorder or infection were excluded. This study was approved by the independent ethics committees at our hospital and was performed in accordance with the ethical standards of the World Medical Association Declaration of Helsinki in 1995 (as revised in Edinburgh 2000). Informed consent was obtained from all included subjects and patient anonymity was preserved. Disease free survival (DFS) was calculated from the date of surgery to local recurrence/distant metastasis, OS was defined as the time interval from the date of diagnosis to death or to the most recent follow-up.

### Treatment

All patients underwent transthoracic esophagectomy with at least a two-field regional lymphadenectomy, including standard, extended, or total dissection of the cervical, thoracic and abdominal lymph nodes, with a median number of dissected lymph nodes of 19 (range, 12-89). Adjuvant chemotherapy or radiotherapy was delivered based on the tumor stage, doctor's selection and patient's treatment desire. Actually, a total of 73 patients received adjuvant chemotherapy, with the aim to decrease the rate of local recurrence and/or distant metastasis. Fluorouracil-based two-drug combination chemotherapy was delivered to four-fifths of the cases, whereas the remaining subjects underwent fluorouracil monotherapy.

### Clinical and laboratory parameters

Patients' baseline information, such as age, gender, weight, height, etc. were retrospectively reviewed and collected from the medical records. Patients were staged based on the AJCC/UICC TNM staging system (the 8th edition). The length of primary tumor and the degree of differentiation were also classified accordingly.

Pretreatment serum albumin level, neutrophil and lymphocyte counts were examined in samples obtained within one week before the initiation of treatment. Serum albumin level was examined by an automatic biochemical analyzer (Roche 501, Japan). Neutrophils and lymphocytes were detected using an XE-2100 automated hematology analyzer (Sysmex Co., Kobe, Japan).

### BMI, ALB and NLR (BAN) score

BAN score was defined as follows:

BAN = (BMI × ALB) / NLR

Where: BMI = weight (kg) / [height (m)]^2^ ; ALB = serum albumin (g/dL); NLR = neutrophil count / lymphocyte count.

In this study, the optimal cutoff value for BAN score was determined with the method established by Jan Budczieset al. at http://molpath.charite.de/cutoff/ 18. Thus, BAN score was classified as low (< 28.0) and high (≥ 28.0) groups in the subsequent analysis.

### Statistical analysis

The difference of baseline characteristics was examined by Chi-square test. Kaplan-Meier method with log-rank test was utilized to estimate survival curves. Prognostic factors were determined by univariate and multivariate analysis with Cox proportional hazards regression models and hazard ratios (HRs) for variables respecting to DFS and OS were also calculated. HRs with 95% confidence intervals (CIs) and two-sided *P* value were reported. All statistical analysis was carried out with SPSS 21.0 (SPSS Inc., Chicago, IL, USA). And a two-sided *P* value of less than 0.05 was considered to be with statistical significance.

## Results

### Patient baseline characteristics

A total of 420 patients were selected in the final analysis. Patients' baseline characteristics were demonstrated in Table 1. The median age was 60.0 years (ranged, 20.0-87.0 years), and 75.7% of the cases were males. Tumors with well differentiation, moderate differentiation and poor/none-differentiation were observed in 107 (25.5%), 213 (50.7%) and 100 (23.8%) patients, respectively. The primary tumor invasion depth of T1, T2, T3, and T4 were found in 40 (9.5%), 66 (15.7%), 280 (66.7%), and 34 (8.1%) of the subjects, respectively. Lymph node was involved in 205 (48.8%) of the patients. And the numbers of subjects diagnosed with stage I, II, and III disease were 24 (9.2%), 127 (48.8%) and 107 (41.9%), respectively (Table 1).

### Correlation between pretreatment BAN score and clinicopathologic parameters

Patients were classified into two groups: those with low (< 28.0, more inflammation) and high (≥ 28.0, less inflammation) BAN scores. The baseline parameters were listed in Table 1. Results showed that pretreatment BAN score was significantly correlated with tumor length, pretreatment WBC count, BMI, ALB and NLR. Whereas no significant difference was seen in age, gender, tumor location, degree of differentiation, depth of invasion, lymph node status, tumor-node-metastasis (TNM) stage, alcohol consumption, smoking or adjuvant treatment between the two groups (Table 1).

### Prognostic value of pretreatment BAN score in resectable ESCC

**Figure 1:**
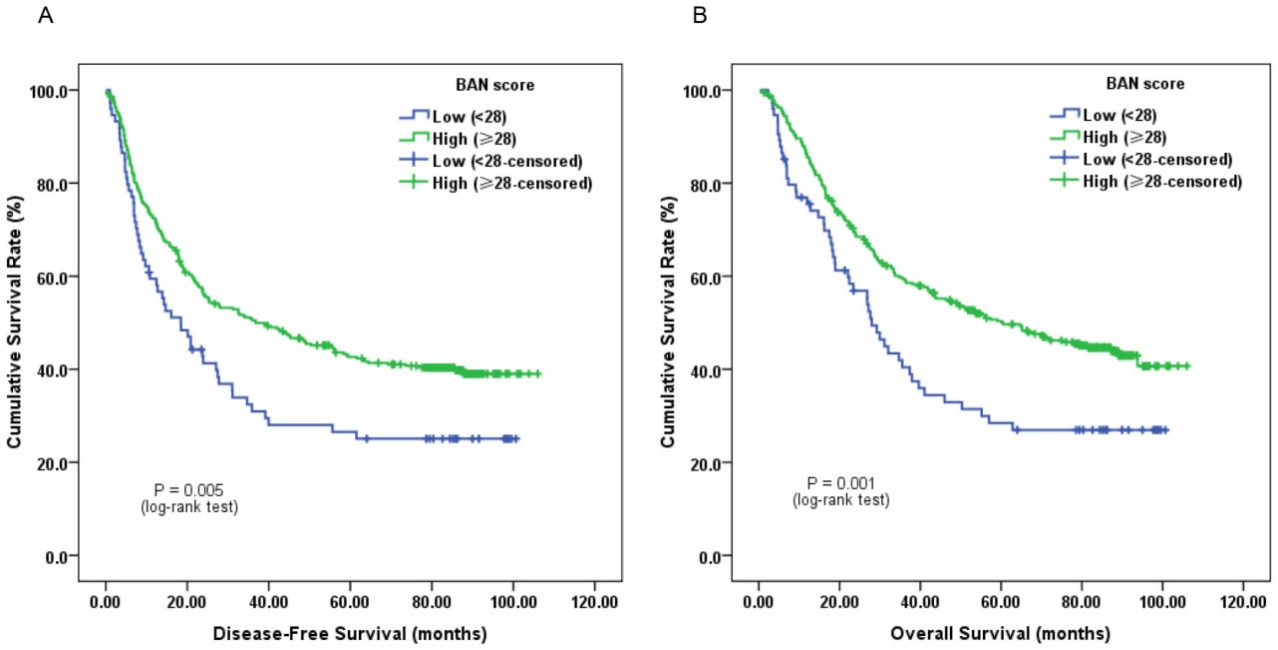
Kaplan-Meier survival curves of A, disease-free survival (DFS) and B, overall survival (OS) stratified by pretreatment body-mass-index, albumin and neutrophil-lymphocyte ratio (BAN) score in 420 esophageal squamous cell carcinoma (ESCC) patients (with log-rank test).

Survival analysis demonstrated that subjects with low pretreatment BAN score (< 28.0, more inflammation) tended to have a worse median DFS of 18.4 months (95% CI, 11.2-25.6 months) and OS of 28.0 months (95% CI, 23.2-32.7 months), respectively. Whereas the median DFS and OS for those with high pretreatment BAN score (≥ 28.0, less inflammation) were 36.8 months (95% CI, 21.4-52.2 months) and 60.2 months (95% CI, 40.9-80.0 months), respectively. Log-rank test showed that both DFS and OS were significantly different between the two groups (*P* = 0.005 and *P* = 0.001, respectively) (Figure 1).

In univariate analysis of DFS, the results revealed that low pretreatment BAN score was significantly associated with impaired DFS (HR, 0.650; 95% CI, 0.481-0.877; *P* = 0.005; Figure 1A). Tumor length (<5 vs. ≥5 cm), depth of invasion (T1-2 vs. T3-4), lymph node involvement (Negative vs. Positive), TNM stage (I-II vs. III), alcohol consumption (Never vs. Ever), smoking (Never vs. Ever) and adjuvant treatment (No vs. Yes) were classified as other significant prognostic indicators (*P* < 0.05). In addition, pretreatment BAN score (HR, 0.726; 95% CI, 0.532-0.993; *P* = 0.045) could independently predict DFS identified by multivariate analysis (Table 2).

Univariate analysis of OS suggested that cases with low pretreatment BAN score tended to have worse OS (HR, 0.608; 95% CI, 0.445-0.829; *P* = 0.002; Figure 1B). Besides, tumor length, depth of invasion, lymph node involvement, TNM stage, alcohol consumption and adjuvant treatment could also significantly predict OS. Moreover, multivariate cox regression analysis found that pretreatment BAN score could also serve as a significant predictor for OS. BAN score of ≥ 28.0 had a HR of 0.670 (95% CI, 0.485-0.927; *P* = 0.016) for OS (Table 3).

**Figure 2:**
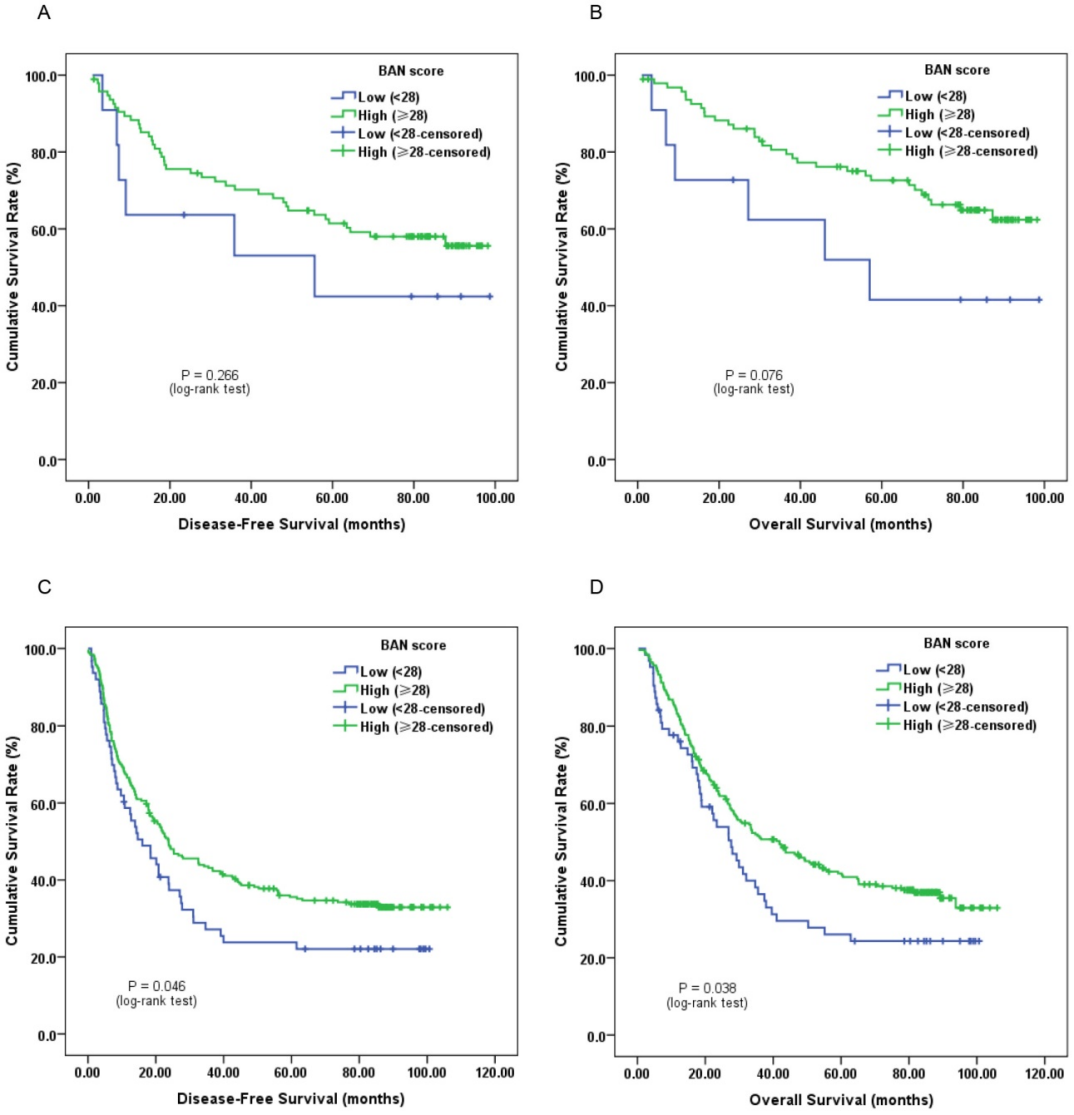
Kaplan-Meier survival curves of A, DFS and B, OS stratified by pretreatment BAN score in T1-2 stage ESCC patients (N = 106); C, DFS and D, OS stratified by pretreatment BAN score in T3-4 stage ESCC patients (N = 314) (with log-rank test).

**Figure 3:**
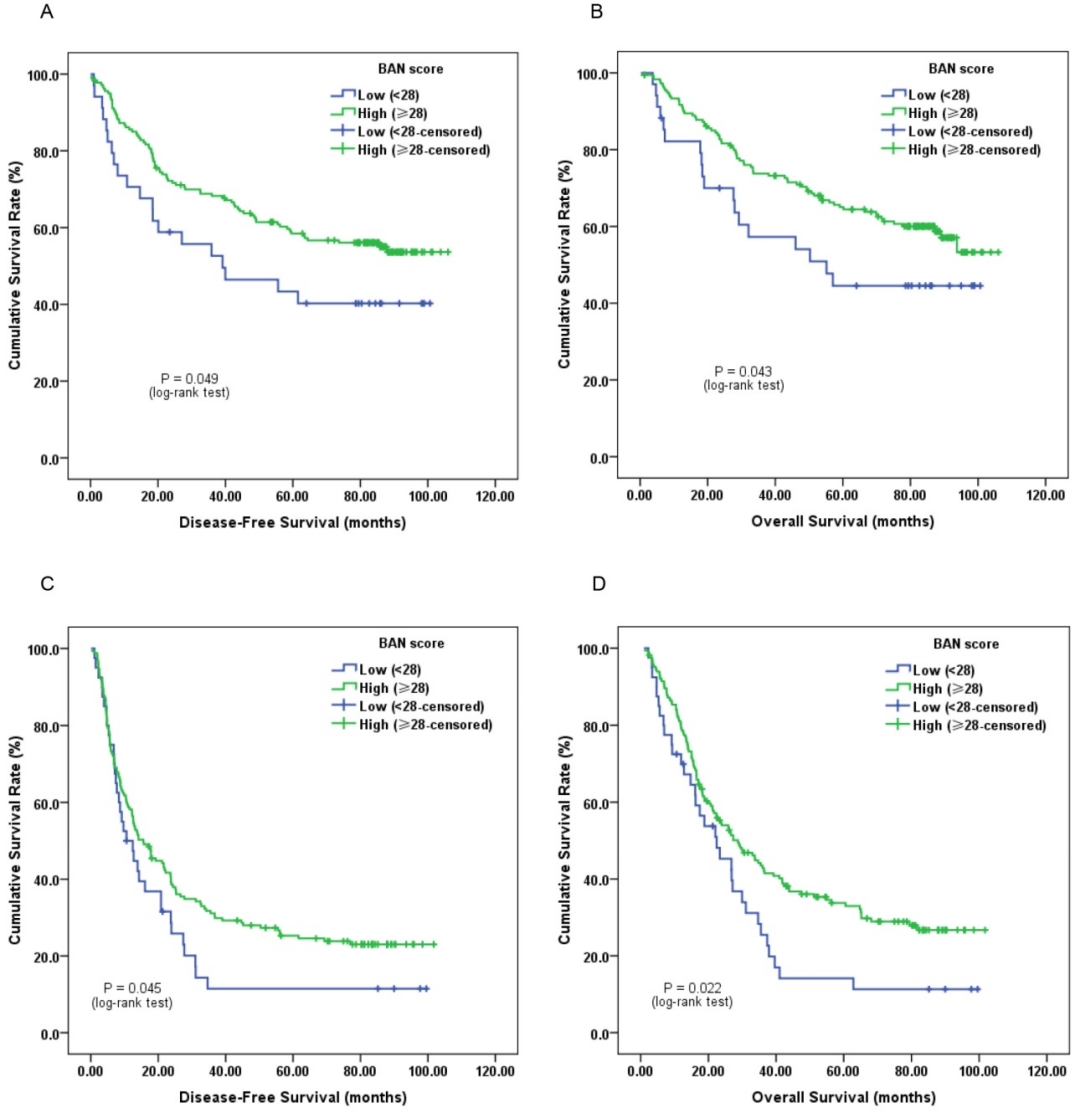
Kaplan-Meier survival curves of A, DFS and B, OS stratified by pretreatment BAN score in ESCC patients without lymph node involvement (N = 215); C, DFS and D, OS stratified by pretreatment BAN score in ESCC patients with lymph node involvement (N = 205) (with log-rank test).

Furthermore, subgroup analysis classified by depth of invasion and lymph node status indicated that low pretreatment BAN score was significantly associated with worse DFS (Figure 2C, 3A and 3C; *P* < 0.05) and OS (Figure 2D, 3B and 3D; *P* < 0.05) in certain cases, but not DFS in T1-2 stage ESCC subjects (Figure 2A and 2B; *P* > 0.05).

## Discussion

Systemic inflammation response has been identified to be involved in the development, progression and distant metastasis, and associated with unfavorable survival in a broad range of malignant diseases, including ESCC 4-7. To the best of our knowledge, this study revealed for the first time that patients with low pretreatment BAN score had more advanced and progressive diseases, and were significantly correlated with worse prognosis. The present study shed light on the importance of evaluating systemic inflammation response in ESCC patients as a prognostic marker.

Clinically, serum CRP, white blood cell, neutrophil and lymphocyte counts are the most commonly measured parameters to evaluate the degree of systemic inflammation response in cancer patients 7. Besides, hypoalbuminemia is also recognized to play vital roles in systemic inflammatory response 11. Moreover, combinations of such variables have been proposed to establish several inflammation-based prognostic formulas.

On the one hand, the modified GPS is determined based on serum CRP and albumin concentrations, and is widely used as a valuable and promising prognostic marker in various malignancies, including ESCC 19-22. However, Tian et al. failed to identify modified GPS as an independent prognostic indicator in 260 ESCC patients who received transthoracic esophagectomy 22. Neither did Arigami et al. found its independent prognostic significance in 238 ESCC subjects who underwent esophagectomy with lymphadenectomy 16. On the other hand, the NLR, a marker of systemic inflammation has also been found to predict unfavorable survival in cases with different cancers including ESCC 14-15. Results from Chen MF' study showed that an elevated pretreatment NLR was significantly associated with advanced-stage disease and impaired OS in 926 ESCC patients who underwent concomitant chemoradiotherapy, but not in the 242 subjects treated with surgical intervention 15. Recently, Arigami T and his colleagues suggested that NLR could serve as a valuable indicator for predicting disease progression and prognosis in patients with ESCC who received esophagectomy with lymphadenectomy 16.

Furthermore, to the best of our knowledge, weight loss was also associated with ongoing systemic inflammation response 23-24. Therefore, we established BAN score, which was calculated based on BMI, albumin and NLR, to assess the ongoing systemic inflammation response in these patients. Subjects with BAN score of < 28.0 indicating high systemic inflammation response were found to have more advanced tumor length. However, no significant difference was observed in BAN score based on age, gender, tumor location, degree of differentiation, depth of invasion, lymph node status or tumor-node-metastasis, suggesting that both groups could generate similar degree of systemic inflammation response. In addition, the multivariate analysis revealed that depth of invasion, lymph node metastasis, alcohol consumption and pretreatment BAN score were independently associated with impaired long-term survival. Of these, depth of invasion, lymph node metastasis and alcohol consumption were non-modifiable variables. Thus, high systemic inflammation response as manifested by ALI < 28.0 provided a novel and prominent therapeutic area for improving the outcome of such cases.

Although the main limitations of this study were the retrospective and single institution design, the findings suggested that pretreatment BAN score could significantly predict long-term survival, help risk stratification more accurately and design optimal therapeutic strategies for resectable ESCC.

## Figures and Tables

**Table 1:** Patient baseline characteristics, clinicopathological features, and pretreatment BAN score (N=420)

Clinicopathologic characteristics	Patients	BAN score (No., %)		*P* value
	No. (%)	Low (< 28.0)	High (≥ 28.0)	
**Age (years)**				0.435
< 60	244 (58.1)	46 (62.2)	198 (57.2)	
≥ 60	176 (41.9)	28 (37.8)	148 (42.8)	
**Gender**				0.772
Male	318 (75.7)	57 (77.0)	261 (75.4)	
Female	102 (24.3)	17 (23.0)	85 (24.6)	
**Tumor location**				0.476
Upper	37 (8.8)	6 (8.1)	31 (9.0)	
Middle	260 (61.9)	42 (56.8)	218 (63.0)	
Lower	123 (29.3)	26 (35.1)	97 (28.0)	
**Tumor length (cm)**				< 0.001^*^
< 5	226 (53.8)	20 (27.0)	206 (59.5)	
≥ 5	194 (46.2)	54 (73.0)	140 (40.5)	
**Differentiation**				0.461
Well	107 (25.5)	22 (29.7)	85 (24.6)	
Moderate	213 (50.7)	38 (51.4)	175 (50.6)	
Poor/Undifferentiated	100 (23.8)	14 (18.9)	86 (24.8)	
**T stage**				0.087
T1	40 (9.5)	3(4.1)	37 (10.7)	
T2	66 (15.7)	8 (10.7)	58 (16.8)	
T3	280 (66.7)	54 (73.0)	226 (65.3)	
T4	34 (8.1)	9 (12.2)	25 (7.2)	
**N stage**				0.518
N0	215 (51.2)	34 (45.9)	181 (52.3)	
N1	111 (26.4)	20 (27.0)	91 (26.3)	
N2	73 (17.4)	17 (23.0)	56 (16.2)	
N3	21 (5.0)	3 (4.1)	18 (5.2)	
**TNM stage**				0.116
I	38 (9.0)	3 (4.1)	35 (10.1)	
II	192 (45.7)	31 (41.9)	161 (46.5)	
III	190 (45.3)	40 (54.0)	150 (43.4)	
**Alcohol consumption**				0.788
Never	278 (66.2)	45 (60.8)	233 (67.3)	
Ever	142 (33.8)	29 (39.2)	113 (32.7)	
**Smoking**				0.855
Never	155 (36.9)	28 (37.8)	127 (36.7)	
Ever	265 (63.1)	46 (62.2)	219 (63.3)	
**Adjuvant treatment**				0.963
No	347 (82.6)	61 (82.4)	286 (82.7)	
Yes	73 (17.4)	13 (17.6)	60 (17.3)	
Pretreatment WBC level (10^^9^/L)	7.5 ± 2.4	9.2 ± 3.2	7.1 ± 2.0	< 0.001^*^
Pretreatment BMI (kg/m^2^)	22.1 ± 3.2	20.5 ± 3.4	22.5 ± 3.0	< 0.001^*^
Pretreatment albumin level (g/L)	43.8 ± 8.1	40.2 ± 5.0	44.5 ± 8.4	< 0.001^*^
Pretreatment NLR level	2.5 ± 2.1	5.0 ± 4.1	1.9 ± 0.6	< 0.001^*^

BAN, BMI-Albumin-NLR; ESCC, esophageal squamous cell carcinoma; TNM, tumor-node-metastasis; WBC, white blood cell; BMI, body mass index; NLR, neutrophil lymphocyte ratio. *P < 0.05.

**Table 2:** Clinicopathological factors, BAN score, and DFS: univariate and multivariate analysis (N=420)

Variables	Univariate			Multivariate		
	HR	95% CI	*P*	HR	95% CI	*P*
**Age (years)**						
< 60 vs. ≥ 60	1.024	0.801-1.309	0.852			NI
**Gender**						
Male vs. Female	0.748	0.555-1.007	0.056			NI
**Tumor location**						
Middle vs. Non-middle	0.987	0.797-1.218	0.890			NI
**Tumor length (cm)**						
< 5 vs. ≥ 5	1.504	1.179-1.920	0.001^*^	1.095	0.843-1.421	0.498
**Differentiation**						
Moderate/Well vs. Poor/Undifferentiated	1.111	0.839-1.472	0.462			NI
**Depth of invasion**						
T1-T2 vs. T3-T4	2.115	1.536-2.911	< 0.001^*^	1.508	1.072-2.123	0.018^*^
**Lymph node involvement**						
Negative vs. Positive	2.634	2.045-3.392	< 0.001^*^	2.192	1.670-2.878	< 0.001^*^
**TNM stage**						
I-II vs. III	2.248	1.755-2.880	< 0.001^*^			NI
**Alcohol consumption**						
Never vs. Ever	1.408	1.094-1.812	0.008^*^	1.139	0.851-1.524	0.381
**Smoking**						
Never vs. Ever	1.328	1.024-1.724	0.033^*^	1.117	0.828-1.506	0.468
**Adjuvant treatment**						
No vs. Yes	1.561	1.156-2.109	0.004^*^	1.191	0.875-1.622	0.266
**BAN score**						
Low (< 28.0) vs. High (≥ 28.0)	0.650	0.481-0.877	0.005^*^	0.726	0.532-0.993	0.045^*^

DFS, disease-free survival; HR, hazard ratio; CI, confidence interval; NI, not included. *P < 0.05.

**Table 3:** Clinicopathological factors, BAN score, and OS: univariate and multivariate analysis (N=420)

Variables	Univariate			Multivariate		
	HR	95% CI	*P*	HR	95% CI	*P*
**Age (years)**						
< 60 vs. ≥ 60	1.134	0.878-1.465	0.336			NI
**Gender**						
Male vs. Female	0.770	0.566-1.047	0.096			NI
**Tumor location**						
Middle vs. Non-middle	0.957	0.765-1.196	0.698			NI
**Tumor length (cm)**						
< 5 vs. ≥ 5	1.429	1.107-1.844	0.006^*^	0.980	0.745-1.290	0.888
**Differentiation**						
Moderate/Well vs. Poor/Undifferentiated	1.103	0.823-1.478	0.512			NI
**Depth of invasion**						
T1-T2 vs. T3-T4	2.393	1.690-3.389	< 0.001^*^	1.804	1.248-2.607	0.002^*^
**Lymph node involvement**						
Negative vs. Positive	2.602	1.996-3.391	< 0.001^*^	2.135	1.611-2.829	< 0.001^*^
**TNM stage**						
I-II vs. III	2.320	1.789-3.008	< 0.001^*^			NI
**Alcohol consumption**						
Never vs. Ever	1.521	1.170-1.978	0.002^*^	1.338	1.023-1.748	0.033^*^
**Smoking**						
Never vs. Ever	1.294	0.987-1.698	0.062			NI
**Adjuvant treatment**						
No vs. Yes	1.577	1.153-2.156	0.004^*^	1.189	0.864-1.635	0.288
**BAN score**						
Low (< 28.0) vs. High (≥ 28.0)	0.608	0.445-0.829	0.002^*^	0.670	0.485-0.927	0.016^*^

OS, overall survival. *P < 0.05.
